# Mechanism of Spontaneous Surface Modifications on Polycrystalline Cu Due to Electric Fields

**DOI:** 10.3390/mi12101178

**Published:** 2021-09-29

**Authors:** Kristian Kuppart, Simon Vigonski, Alvo Aabloo, Ye Wang, Flyura Djurabekova, Andreas Kyritsakis, Veronika Zadin

**Affiliations:** 1 Institute of Technology, University of Tartu, Nooruse 1, 50411 Tartu, Estonia; kristian.kuppart@ut.ee (K.K.); simvig@gmail.com (S.V.); alvo.aabloo@ut.ee (A.A.); ye.wang@ut.ee (Y.W.); andreas.kyritsakis@ut.ee (A.K.); 2 Helsinki Institute of Physics and Department of Physics, University of Helsinki, P.O. Box 43 (Pehr Kalms gata 2), FI-00014 Helsinki, Finland; flyura.djurabekova@helsinki.fi

**Keywords:** nanocrystalline metals, surface diffusion, vacuum breakdowns, molecular dynamics

## Abstract

We present a credible mechanism of spontaneous field emitter formation in high electric field applications, such as Compact Linear Collider in CERN (The European Organization for Nuclear Research). Discovery of such phenomena opens new pathway to tame the highly destructive and performance limiting vacuum breakdown phenomena. Vacuum breakdowns in particle accelerators and other devices operating at high electric fields is a common problem in the operation of these devices. It has been proposed that the onset of vacuum breakdowns is associated with appearance of surface protrusions while the device is in operation under high electric field. Moreover, the breakdown tolerance of an electrode material was correlated with the type of lattice structure of the material. Although biased diffusion under field has been shown to cause growth of significantly field-enhancing tips starting from initial nm-size protrusions, the mechanisms and the dynamics of the growth of the latter have not been studied yet. In the current paper we conduct molecular dynamics simulations of nanocrystalline copper surfaces and show the possibility of protrusion growth under the stress exerted on the surface by an applied electrostatic field. We show the importance of grain boundaries on the protrusion formation and establish a linear relationship between the necessary electrostatic stress for protrusion formation and the temperature of the system. Finally, we show that the time for protrusion formation decreases with the applied electrostatic stress, we give the Arrhenius extrapolation to the case of lower fields, and we present a general discussion of the protrusion formation mechanisms in the case of polycrystalline copper surfaces.

## 1. Introduction

The planned Compact Linear Accelerator (CLIC) [1] in CERN uses extremely high electric fields (~100 MV/m) to accelerate electrons and positrons to energies up to 3 TeV. Operation under such conditions leads to vacuum breakdowns in the accelerating structures [2], decreasing the luminosity of the collider and increasing the power loss. Moreover, the breakdowns modify the surface morphology, which has an overall detrimental effect on the high-precision shape of the metal surfaces of the accelerating structures.

Developing a method to reduce the frequency of vacuum breakdowns requires understanding of origins of the phenomenon. Emission current measurements in alternating current [3] and direct current [4,5] regimes point to the existence of significant field enhancement in the range of *β* ≈ 30–150, the physical origins of which is not entirely clear. It has been theorized [6] that the source for this field enhancement is rough static features residing on the electrode surface acting as field emitters, although surface features with aspect ratio high enough to cause field enhancement of this magnitude have never been observed [5]. Other surface features, such as contaminants, oxides and residual gas are believed to be removed from the electrodes subject to the conditioning process [4] in CLIC and thus not crucial to the breakdown phenomenon. One additional partial explanation for this field enhancement can be given by Schottky’s conjecture—the resulting field enhancement from a field emitter imposed on top of another emitter is approximately the product of the respective field enhancements, given that the dimensions of the emitters differ by approximately one order of magnitude [7,8].

While static field emitters on the surface have not been observed, it has been hypothesized, that these field emitters can dynamically appear by enhanced dislocation activity [1,9] and surface self-diffusion, due to the applied electrostatic stress, Joule heating due to emission currents [10], electromigration and other phenomena [11,12], leading to a self-reinforcing process.

Studies of different materials show that there is a clear correlation between breakdown field strength and crystal structure [2]. Additionally, it has been found [5] that the quantity βE depends only on the electrode material, where E is the macroscopic mean electric field. Thus, a hypothesis can be formulated that the onset of breakdown is microscopic in nature and that studying the atomistic and crystallographic behavior of materials under high electric fields is a worthwhile endeavor. Because of the difficulty of conducting atomic level microscopy experiments in high field conditions, computer simulations become a viable means of investigating the accelerator materials. The role of dislocations in surface modification has been studied in simulations of monocrystalline materials. Because of the high stress required to create dislocations in a perfect crystal, various imperfections have been introduced into the simulations. Simulations of subsurface voids [1] and precipitates [13] have shown that these sites can generate plateaus or protrusions on the surface when sufficient external stress is applied.

Nanocrystalline materials have also been studied by simulations. For example, simulations of bulk nanocrystalline copper [14,15] have shown that the microscopic plastic deformation mechanism changes from a dislocation based mechanism to grain boundary sliding at grain sizes of 10–15 nm, where the yield strength is maximal. At grain sizes in the grain boundary sliding regime, most of new dislocations are generated at the grain boundaries. The effect of tensile stress in a copper nanocrystal parallel to the surface has been explored in ref. [16]. It was found, that the increasing stress increases the surface roughness considerably and gives rise to the appearance of surface protrusions.

Recent kinetic Monte Karlo simulations [17] have shown that starting from surface protrusions of a few nm size, larger scale field-enhancing tips can grow due to the biased diffusion effect [18,19]. However, the dynamics and the possible mechanisms behind the formation of the initial nano-protrusions has not been explored yet. Keeping in mind that the diffusion coefficient at grain boundaries has been found to be much higher than in the bulk of the crystal [20,21], in this paper we explore self-diffusion on the grain boundaries as a possible mechanism for the spontaneous formation of nanometer-size protrusions. We use molecular dynamics (MD) simulations to study the effect of the external stress induced by an electric field on the surface morphology of nanocrystalline copper and we investigate possible surface protrusion growth mechanisms due to the enhanced mobility of atoms on surface grain boundaries. Protrusion formation is analyzed under different conditions of stress and temperature, leading to an estimation of the formation time under normal CLIC operating conditions. 

## 2. Methods

The MD simulations were conducted using classical MD code LAMMPS [22]. An Embedded Atom Method (EAM) potential [23] by Mishin et al. [24] is used to model the Cu interatomic forces, which has shown good properties for simulating surfaces. In the EAM model, the potential energy of the system is given by:

(1) $$E=\frac{1}{2}\underset{ij}{\sum }V\left(r_{ij}\right)+\underset{i}{\sum }F\left(\rho_{i}\right)$$

where $V\left(r_{ij}\right)\;$ is the pair-potential term and $F\left(\rho_{i}\right)$ is an embedding energy term as a function of the electron density at the atom site i, stemming from neighboring atoms. The velocity-Verlet algorithm with a timestep of Δt=2 fs was used throughout this work to propagate the positions and velocities of the atoms. 

The most significant effect of an external electric field on a metal surface is that it exerts an a tensile stress to the material surface, i.e. the Maxwell stress

(2) $$\sigma =\frac{\varepsilon_{0}}{2}F^{2}$$

In Equation (2), F is the electric field strength at a surface point and $\varepsilon_{0}$ is the vacuum permittivity. Although more elaborate hybrid Electrodynamic-MD models are available for including electric field effects in MD [25,26,27], for the purposes of this work it is sufficient to include σ directly in MD as an external force term on the surface atoms. This is valid under the assumptions of a uniform field strength above the surface, which is accurate as long as the local surface curvature is small. The results can be considered valid until significant surface deformation takes place.

Multiplying the stress by the surface area and dividing by the number of surface atoms gives the force acting on a single surface atom:

(3) f=σS/N

This force is added in the MD algorithm to the forces stemming from the neighboring atoms. As a result, surface atoms are pulled away from the surface. Surface deformation is distinguished from the uniform surface strain by comparing the maximum z-coordinate of the surface atoms to the mean z-coordinate of the surface atoms:

(4) δ=max(z)−mean(z)

This characteristic is averaged within a time window of 200 timesteps to decrease noise and the resulting difference is a measure of the maximal surface deformation. A rapid increase in δ indicates the onset of a strong surface deformation and the growth of a protrusion on the surface. It also marks the end of the validity of the uniform electrostatic stress approximation. When δ exceeds 1 nm, the simulation is stopped. We consider the applied stress at that point to be sufficient to induce significant surface deformation and we define it as the critical stress $\sigma_{c}$.

### 2.1. Classification of Surface Atoms 

In the current study, atoms are classified to belong to the surface with the help of coordination analysis. The physical intuition behind using coordination numbers is simple – the surface atoms have, on average, half the neighbors of bulk atoms. To be able to track the surface even in the case of deformed geometries, the neighboring sphere radius should be taken as large as possible. Due to LAMMPS’s implementation for calculating the number of neighboring atoms, this radius cannot exceed the cut-off value for the given interatomic potential and is chosen to be 5 Å, based on the considerations presented below.

Next, we determine a threshold number of neighbors within this radius, which is used to classify the surface atoms. This allows us to determine the atoms that are pulled by the external applied electric field (or the corresponding applied force as it is implemented in our simulations) at any given time, as well as dynamically track the surface deformation during the simulation by calculating changes in the positions of the surface atom. 

**Figure 1:**
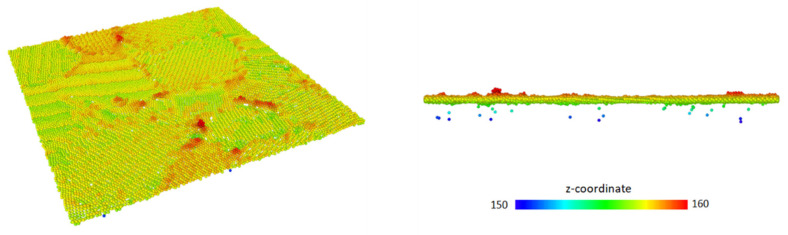
Perspective and 2D view of the resulting surface in the case of deformed geometry at 900 K. Atoms are color coded by the z-coordinate. A small number of low coordination false positives can be seen in the rightmost graph.

To determine the threshold coordination number, three aspects must be considered. Firstly, it is well established [28] that a static electric field only penetrates into the first few atomic layers and thus the algorithm has to consider only the top few surface layers. Secondly, the detected surface should not contain any artificial holes. Thirdly, the amount of low coordination atoms in the bulk classified as surface atoms should be minimal. Taking these restrictions into account, the reasonable neighboring sphere radius is set to 5 Å and the threshold number of neighbors within that sphere to 37. Sensitivity test simulations showed that within a coordination number in the range of 37 ± 3, the critical stress differs by less than 5%, which is comparable to the statistical uncertainty of the simulations. One example of the resulting surface in deformed state is shown Figure 1**.** We can see the existence of small number of wrongly identified surface atoms in the bulk. The number of such atoms decreased further at lower temperatures and in the case of undeformed surfaces. 

To give a worst-case order-of-magnitude estimate of the effect of the external force on bulk atoms classified as surface atoms, we make the following considerations. First, as the classification of surface atoms is updated every 20 timesteps, we consider the artificial force acting only for this duration. Second, as the number of false positives at temperatures below 900 K is negligible, we use the force calculated from the critical stress at this temperature. Thus we can calculate the additional kinetic energy obtained by an atom in this duration:

(5) $$\Delta E_{k}=\frac{\Delta p^{2}}{2m}=\left(\frac{f^{2}\Delta t^{2}}{2m}\right)=1.2\;\mathrm{meV}$$

where f≈1 eV/nm is the force on surface atom corresponding to critical stress value at 900 K, Δt=40 fs, and *m* is the mass of a copper atom. We can compare this energy to the vacancy formation energy for this potential: $E_{f}=1.27\;\mathrm{eV}$. We can see, that this energy is 3 orders of magnitude greater than the additional energy provided by the artificial force. Hence we can conclude that these artifacts cannot influence significantly the simulation outcome.

### 2.2. Generation and Preparation of Simulated Polycrystal

The initial polycrystalline structures were generated with Atomsk [29] using a Voronoi tessellation, which has been previously used to create polycrystalline models [14]. We created 20 different randomized polycrystalline configurations with dimensions of 30 × 30 × 15 nm, containing approximately 1.14 million atoms. Half of them contained 9 grains, corresponding to a mean grain diameter of 13.8 nm, while the other half contained 18 grains, corresponding to a mean grain diameter of 11.0 nm. 

Simulations were conducted in two parts, preparation and force ramping. The same simulation procedure was repeated for each of the 20 configurations to obtain a statistical sample of surface processes and to be able to mimic a larger polycrystal configuration. Visualization of atomic configurations and post-processing are done using OVITO [30].

The systems were initially generated as fully periodic. However, since the Voronoi tessellation procedure can lead to structures far from equilibrium, an energy minimization algorithm has to be employed to relax the obtained structures. For that effect, the Polak-Ribiere non-linear conjugate gradient (CG) minimization scheme [31] with relative energy tolerance of $10^{-8}$ was used. The simulation box was allowed to relax possible external pressure components, so that $p_{xx}=p_{yy}=p_{zz}=0$. An average of 5000 iterative solver steps was required to achieve the required tolerance. 

**Figure 2:**
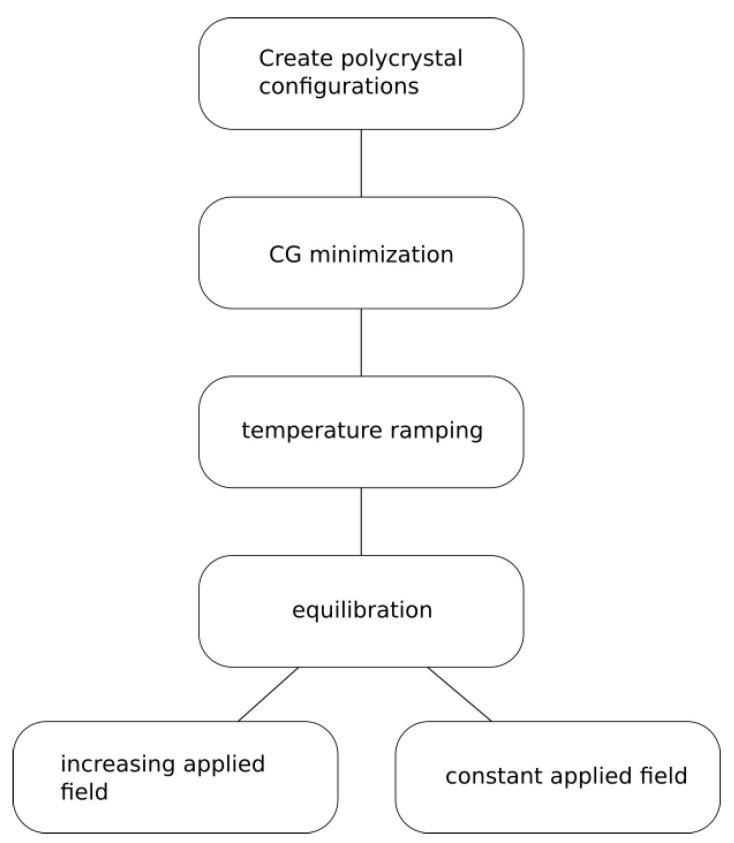
Schematic workflow of the simulation process. 20 polycrystalline structures were obtained after the preparation process. Increasing applied field simulations were conducted for each structure at 7 different temperatures. Constant applied field simulations were conducted for a specific structure at 900 K for 10 fractional values of the critical stress. Each of those were repeated twice to obtain adequate statistics.

After CG relaxation was finished, the systems were considered to be in an equilibrium state for 0 K. To heat the systems up to the temperature used in the simulations, initial velocities of the atoms corresponding to 10 K were randomly generated and the temperature was increased using the NPT ensemble implemented in LAMMPS, i.e. the Nose-Hoover [32] thermostat with a time constant of 0.1 ps and the Nose-Hoover barostat with a time constant of 1 ps, in order to keep zero external pressure and allow for thermal expansion. For each polycrystalline sample, 7 different target temperatures from 300 K to 1200 K were obtained using this approach. Finally, the box size in the z-direction was increased so that two free surfaces formed and the system was further relaxed for 200 ps with fixed periodic boundaries in the x and y directions at the target temperature, resulting in surface contraction due to surface tension. As a result of such procedure, the initial states of a polycrystalline copper with open surface were prepared so that surface stress effects were correctly accounted for at given target temperatures. An overview of the preparation and simulation workflow is given in Figure 2.

### 2.3. Simulations with Electric Fields

#### 2.3.1. Simulations with Ramped Electric Fields

The applied stress was linearly ramped from 0 Pa with a rate of 0.024 GPa/ps. The ramping is necessary to avoid the generation of a strong shockwave through the material due to a sudden application of a high tensile stress. The 3 bottom layers of atoms were fixed to mimic a semi-infinite bulk material. During ramping, the Langevin thermostat [33] with a time constant of γ=0.1 ps was applied to the bulk of the system excluding the surface region, to avoid modifying the relevant dynamics. The Langevin thermostat introduces a source of randomness into the system, which enables statistical sampling of the same configuration with same starting conditions.

#### 2.3.2. Simulations with Constant Applied Electric Fields

In addition to the simulations with linearly increasing stress, simulations with constant acting stress were also conducted. This simulations were performed with one particular configuration at the temperature of T=900 K. As in the previous case, the temperature was held constant using a Langevin thermostat for all atoms, except the surface and bottom layers. In these simulations, the stress was increased linearly with the same rate as in the previous section, but after the ramping completed, the stress was held constant at a fractional value $\sigma_{f}$ of the critical stress $\sigma_{c}$. To account for statistical uncertainty, four simulations with different random seeds were conducted as described in the previous section and the resulting average critical stress of $\sigma_{c}=3.07\pm 0.05\;\mathrm{GPa}$ was obtained. All other boundary and simulation conditions were the same as in the previous section. Simulations with $\frac{\sigma_{f}}{\sigma_{c}}=0.4,0.45,\ldots 0.95$ were conducted and the simulation time needed to reach a surface deformation with δ=1 nm was recorded. In addition, a total of 4 simulations were conducted for each value of $\sigma_{f}$.

### 2.4. Analysis of Surface Diffusion

#### 2.4.1. Temperature Dependence of Critical Mechanical Stress

To analyze the temperature dependence of the critical stress, we follow the approach outlined in [34]. We assume that the onset of local surface deformation can be characterized as a thermally activated process, with a mean activation energy per atom Q(f), where *f* is the force acting on a surface atom exposed to the electric field. We assume, that this activation energy decreases linearly with the force acting on the atom:

(6) $$Q=Q_{0}-cf$$

where *f* is the force acting on a surface atom and *c* is a characteristic length scale corresponding to a particular process. This approach is similar to [35], where the enthalpy of formation of a defect was expressed as

(7) $$H_{f}=E_{f}-\sigma \Delta V,$$

where $E_{f}$ is the formation energy of the defect, $\sigma =\epsilon_{0}E^{2}/2\;$ is the Maxwell stress acting on the surface and ΔV is the relaxation volume of the defect. 

The surface diffusion coefficient $D_{s}$ follows an Arrhenius type equation [34]:

(8) $$D_{s}=D_{0}\exp\left(-\frac{Q\left(f\right)}{kT}\right).$$

$D_{0}$ is the exponential pre-factor, which can be calculated as $D_{0}=\nu_{0}a^{2}$, with $\nu_{0}$ being the attempt frequency on the order of $10^{12}-10^{13}s$ and a the length of an atomic jump, a≈0.3 nm. We assume that the criterion for the growth of a surface deformation is close to the surface diffusion coefficient $D_{s}$ reaching a value corresponding to surface pre-melting, about $D_{s}=2\cdot 10^{-5}\frac{{\mathrm{cm}}^{2}}{s}$ [34]. Expressing the activation energy in terms of the force acting on the surface atoms and rearranging, we get a linear relationship between the force acting on the surface atoms and the temperature:

(9) $$f=\ln\left(\frac{D_{sc}}{\nu_{0}a^{2}}\right)\frac{k_{b}T}{c}+\frac{Q_{0}}{c}$$

where $D_{sc}$ is the diffusion constant corresponding to surface pre-melting.

#### 2.4.2. Dependence of the Deformation Time with the Force Acting on the Surface Atoms

We assume that the time $\tau_{c}$ for a surface protrusion to form under constant tensile stress depends exponentially on the activation energy for that process:

(10) $$\tau_{c}=\tau_{0}\exp\left(\frac{Q\left(f\right)}{k_{b}T}\right)$$

where $t_{0}$ is the time needed for the protrusion to form in the case of Q(f)=0. We can rewrite the previous equation as:

(11) $$\ln\left(\frac{\tau_{c}}{\tau_{0}}\right)=\frac{Q_{0}}{k_{b}T}-\frac{c}{k_{b}T}f$$

We can see that there should be a linear relationship between $\ln\left(\tau_{c}\right)$ and the force acting on the surface atoms.

## 3. Results and Discussion

### 3.1. Linearly Increasing Stress

**Figure 3:**
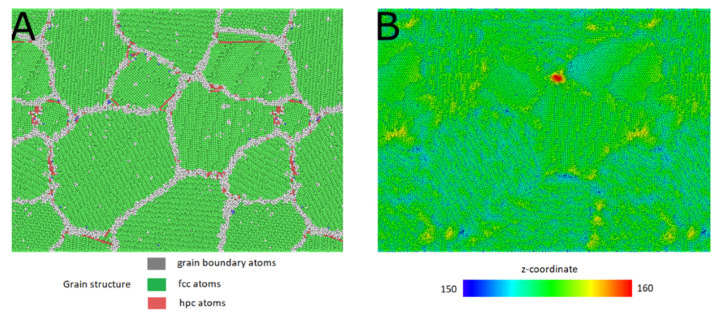
(**A**) Grain structure underlying the surface. Atoms are colored by PTM structure type—green: fcc atoms, grey—grain boundary atoms, red: hcp atoms (atoms part of a stacking fault). (**B**) Height map of the surface in the case of critical surface deformation. Atoms are colored by the z-coordinate. Surface protrusion has formed above a quadruple junction.

**Figure 4:**
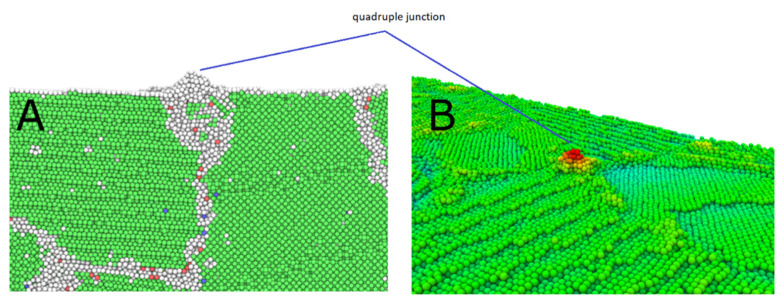
The cross section (**A**) and a perspective view (**B**) of a surface protrusion. Atoms are colored by the PTM parameter. Video S1 of the protrusion formation is given in Supplementary Materials.

One example of the simulated systems is shown in Figure 3. It can be seen that surface deformation due to the applied electric field starts at grain boundaries or triple junctions lying on the surface, in this particular case, from a quadruple junction. This trend was ubiquitous in all of the ≈150 conducted simulations for all simulated configurations and temperatures. Figure 3B shows a height map of the surface at the timestep where a forming protrusion reached the critical height. Figure 3A shows the underlying grain structure at the same location. Atoms are colored using Polyhedral Template Matching (PTM) [36] to reveal their local lattice type. PTM is an analysis method similar to Common Neighbor Analysis [37], but offers a more robust identification of crystal structure at elevated temperatures [36]. Figure 4 depicts the cross section and the perspective view of the same protrusion. It can be seen from these figures that a single protrusion has formed directly above a quadruple junction. While each configuration had preferential sites for the creation of similar protrusions, the exact location differed from simulation to simulation due to stochasticity of the used Langevin thermostat.

**Figure 5:**
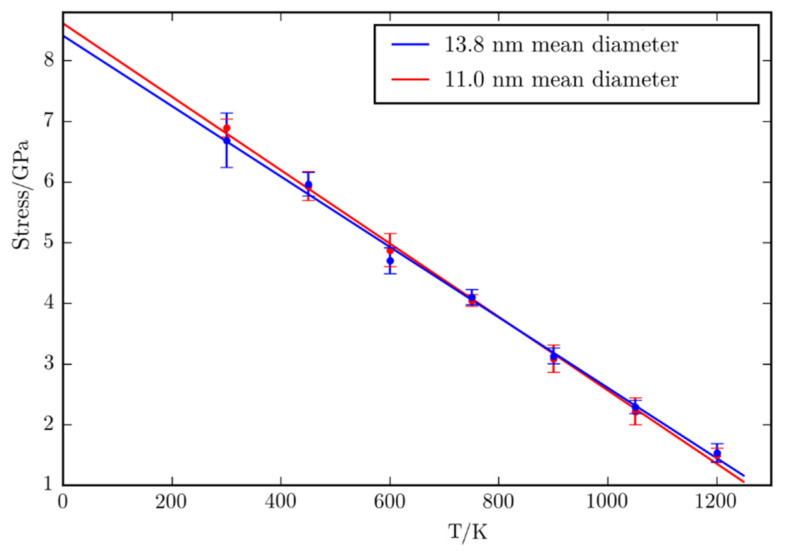
The dependence of critical stress with temperature.

Figure 5 shows the dependence of the critical stress to temperature. Each data point corresponds to the averaged critical stress over 10 different initial geometries with the error bars representing the corresponding standard deviations. It can be seen that the systems corresponding to these mean diameters do not differ significantly and for that reason, in the following only the cases corresponding to mean grain diameter of 13.8 nm are analyzed. It can be seen from the graph that to a good approximation, the stress needed for the surface to reach critical deformation decreases linearly with the temperature. The extrapolated value at 0 K is $\;\;\sigma_{c0}=8.61\;\mathrm{GPa}$.

The slope of the line is $-6.1\cdot 10^{-3}\frac{\mathrm{GPa}}{K}$ and the x-intercept is $T_{c}=1424\;K\;$, slightly higher, than the melting temperature of copper for the used potential [24]. 

To follow the approach outlined in Section 2.4.1, we normalize the stress by the mean number of surface atoms, N≈18,500 and the surface area A=30 nm×30 nm , which is fixed. Doing so we can assess the relevant parameters: $\frac{Q}{c}=2.6\frac{\mathrm{eV}}{\mathrm{nm}}$, $\frac{1}{c}\ln\left(\frac{D_{sc}}{\nu_{0}a^{2}}\right)=-21\frac{1}{\mathrm{nm}}.$ It is natural to assume that c is on the order on one atomic spacing, c≈0.3 nm. Using these results we can calculate the value of the effective activation energy $Q_{0}=0.78\;\mathrm{eV}$ and the surface diffusion coefficient $D_{sc}=1.65\cdot 10^{-5}\frac{{\mathrm{cm}}^{2}}{s}\;$. This activation energy is close to the reported values for the relevant processes, namely surface and grain boundary diffusion [38]. The obtained critical surface diffusion coefficient is close to the reported value for surface pre-melting, which gives some basis for the claim that surface diffusion is the limiting factor in the creation of such surface deformations.

### 3.2. Constant Mechanical Stress

**Figure 6:**
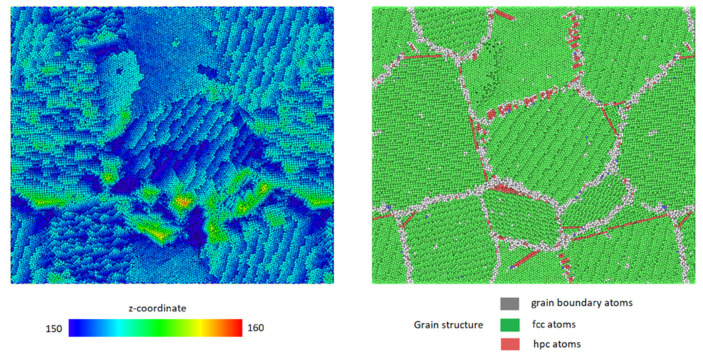
Height mapping (**left**) and the underlying grain structure (**right**) of the system subject to constant tensile stress. A clear tendency of self-roughening is seen under the constant stress, which is lower than critical one in the left image.

To analyze the systems in the case of a constant tensile stress acting on the surface, simulations were conducted as described in Section 2.3.2. Figure 6 shows the height map of the surface and the underlying grain structure of the particular configuration at the timestep corresponding to a surface deformation of height $\delta_{z}=1\;\mathrm{nm}$ and electrostatic stress of $\sigma_{f}=1.5\;\mathrm{GPa}$. This stress is significantly lower than the critical stress of the same system at the same temperature, which implies a strong viscoelastic effect in the simulations under study and hints at the possibility that similar deformations would form under even lower stresses, given an evolution time unobtainable by MD simulations. From Figure 6 it can be seen that longer ridges develop along the surface - grain boundary intersections, unlike in the case of linearly increasing stress, where a single surface deformation spot developed. This shows that there are several sites above which a protrusion can form in each configuration.

**Figure 7:**
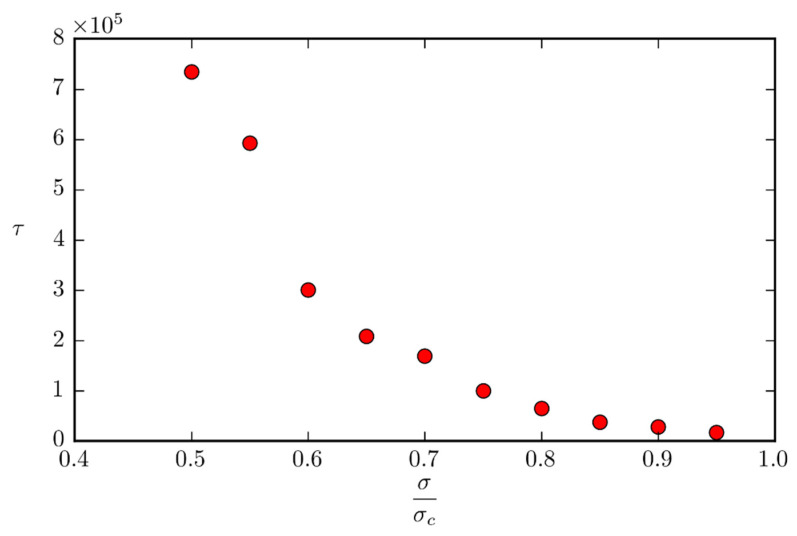
Dependence of the time for a protrusion to form with the force.

The dependence of the time for a protrusion of height $\delta_{z}=1\;\mathrm{nm}$ to form for the given configuration at 900 K is depicted in Figure 7. Each data point corresponds to the average of four simulations. It can be seen from the graph that in the simulated timeframe, critical surface deformation is reached at a surface stress as low as half of the critical stress for that configuration at that temperature.

**Figure 8:**
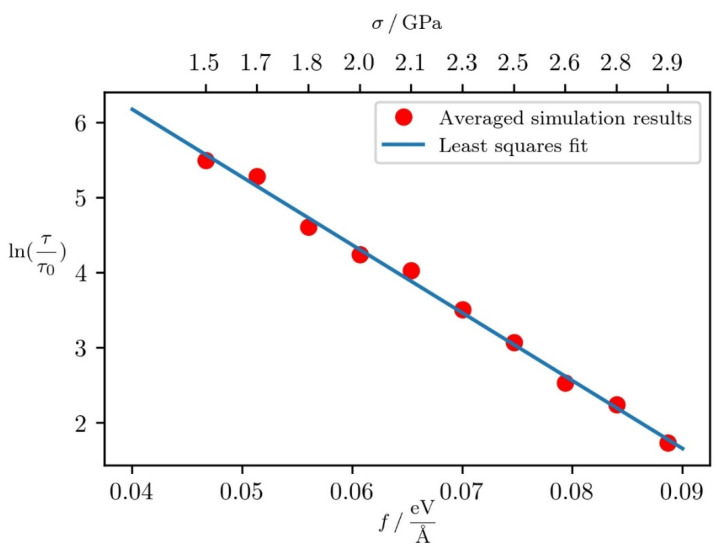
Logarithmic graph of the protrusion formation time under different stress conditions. Each Data point was.averaged over 3 simulations. Least squares fit used to estimate atomic parameters is given by the blue line.

To quantify the creation of such deformations at a constant tensile stress, we use the results of Section 2.4. We estimate the value of $\;\tau_{0}$ to be about 3000 timesteps in these simulations. The exact value of this parameter is not crucial, as only the intercept depends on $\ln\left(\tau_{0}\right)$. A logarithmic plot of the protrusion forming time is given in Figure 8. It can be seen that the graph is linear to a good approximation. From this, we can extract the relevant parameters: $Q_{0}=0.76\;\mathrm{eV}$, $c_{2}=0.7\;\mathrm{nm}$. The force proportionality constant is larger than in the previous case, indicating that in the case of constant tensile stress and longer simulation times, the stress acting on the surface is more effective in giving rise to surface deformations.

## 4. Discussion

**Figure 9:**
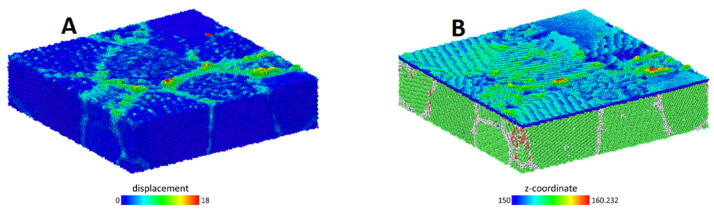
(**A**) Total displacement of atoms until critical surface deformation. (**B**) Height map of the surface and uderlying grain structure. It can be seen that atoms are most mobile on surface and grain boundary intersections. Only a small surface slide is shown for clarity.

In principle, there are 4 different mechanisms contributing to protrusion formation present in our simulations – surface diffusion, surface grain boundary diffusion, diffusion along bulk grain boundaries and intra-grain dislocation activity. Figure 9A shows the total displacement of surface atoms after surface deformation has taken place. From the figure we can qualitatively conclude, that the greatest contribution to protrusion formation stems from diffusion along the intersection of the surface and grain boundaries, also to a lesser extent, along bulk grain boundaries. The rest of the mechanisms seem to have minimal impact on protrusion formation, which allows us to conclude that surface grain boundaries form the primary pathway for surface diffusion. This also explains the results that the protrusion growth preferably starts at grain boundaries and triple junctions. As presented in Figure 4, the protrusion growth site in triple junction locations represents a collection of material structure defects with high scale of disorder. Thus, we can hypothesize that not only triple junctions are responsible for initiation of protrusions, but any kind of surface intercepting large enough structural defect with similar disordered structure can be source of surface protrusions. 

From the quantities calculated in Section 3.2, we can estimate the time needed for a protrusion to form at CLIC operating conditions of E=100 MV/m, T=300 K: τ≈40 s. This time is comparable to the time for the relaxation of similar protrusions as calculated in [39], which means that even at moderate electric field values there should be a possibility for this kind for surface protrusion formation. These mechanisms are further enhanced due to possible pre-enhancement of the electric field by micro-scale surface irregularities and due to increased temperature from emission currents. While we believe that this simple way of accounting for the effect of electrostatic stress on the surface is valid until the onset of surface deformation, it is possible that atomically rough surface protrusions give rise to preliminary field enhancing features, which accelerates their growth due biased field-induced diffusion, leading to a self-reinforcing process that eventually leads to a significant field enhancement, intense field emission, thermal runaway and vacuum breakdown. Development of MD models capable of fully calculating the field-induced biased diffusion on the level of single adatoms are left for future work. 

## 5. Conclusions

We present a spontaneous surface modification mechanism of field emitters, responsible of initiation of initial stages of vacuum breakdown. This phenomena is critical limiting factor in the design of many high electric field devices, such as particle accelerators (CLIC in CERN), free electron lasers, linear accelerators for hadron therapy and many more.

We performed molecular dynamics simulations to study the surface deformation and resulting growth of nanometer-scale protrusions on nanocrystalline copper surfaces under electric field induced stress and we found that nano-protrusions exclusively formed at the intersections of grain boundaries with the surface. This was explained through the increased mobility of surface atoms on grain boundaries, which further underscore the importance of the effect of grain boundaries on the processes on metal surfaces. Thus, as indicated by the structure of the protrusion formation sites, we expect any surface intercepting large enough amorphous nanocluster to be capable of initiating a protrusion growth. How exactly such defects would form or what would cause them remains to be the question of future studies. Moreover, the electrostatic stress needed to trigger a protrusion formation was found to linearly decrease with the temperature of the system which implies that elevated temperatures, for example from strong local heating from field emission currents, further reinforce the process of such emitter formation. The time for protrusion growth under constant electrostatic stress was found to follow the Arrhenius form, exponentially growing with the lowering of acting stress. The time necessary for a protrusion growth in the low field limit (E=100 MV/m, T=300 K) was estimated to be in order of magnitude less than 100 seconds, making it comparable to the relaxation time of similar protrusions as calculated in [39]. Thus, even at moderate electric field values the formation of such surface protrusion becomes expectable. Moreover, once the protrusion growth is initiated, positive feedback loops, as reported in [17,39], can become active, finally leading to the breakdown events.

## Supplementary Materials

The following are available online at https://www.mdpi.com/article/10.3390/mi12101178/s1, Video S1: dynamic-formation-of-protrusion.mp4.
